# Intrinsic Versus Extrinsic Sinus and Atrioventricular Nodes Dysfunction in Athletes: Insights From Transesophageal Electrophysiological Testing With Autonomic Blockade

**DOI:** 10.31083/RCM42738

**Published:** 2025-11-27

**Authors:** Sergei Bondarev, Domenico Corrado, Alessandro Zorzi

**Affiliations:** ^1^Lesgaft National State University of Physical Education, Sports and Health, 190121 St. Petersburg, Russia; ^2^State University of Paediatric Medicine of St. Petersburg, 194100 St. Petersburg, Russia; ^3^Department of Cardiac, Thoracic, Vascular Sciences and Public Health, University of Padua, 35128 Padua, Italy

**Keywords:** cardiac conduction system, electrophysiological study, epigenetics, ion channel remodelling, sports cardiology

## Abstract

**Background:**

While sinus bradycardia and atrioventricular (AV) block in athletes have traditionally been viewed as benign consequences of enhanced vagal tone, recent evidence suggests that, in some individuals, nodal dysfunction may be intrinsic and potentially mediated by epigenetic mechanisms. Therefore, differentiating between these mechanisms is crucial for guiding appropriate clinical management.

**Methods:**

Among 550 elite athletes undergoing routine cardiovascular evaluation, 72 were referred for a transesophageal electrophysiological study (EPS): 58 with significant sinus bradycardia or suspected AV node dysfunction (cases) and 14 athletes with symptoms consistent with supraventricular tachyarrhythmias but no bradyarrhythmia (controls). All participants underwent an EPS to assess corrected sinus node recovery time (CSNRT) and AV nodal Wenckebach point. In the case group, 24 athletes exhibited abnormal parameters at baseline and underwent a repeat EPS following complete autonomic blockade with intravenous propranolol and atropine, aimed at suppressing extrinsic autonomic influences.

**Results:**

The corrected sinus node recovery time exceeded 550 ms in 18 (31%) cases, and the Wenckebach point was greater than 500 ms in 8 (14%) cases. In all eight athletes with baseline AV conduction abnormalities, they normalized after autonomic blockade, consistent with a functional vagal mechanism. In contrast, the mean sinus rate remained unchanged after autonomic blockade, and in 12/18 (67%) of the athletes with prolonged CSNRT, continued to exhibit abnormal values despite autonomic suppression, indicating a probable intrinsic origin. Control subjects showed normal EPS parameters.

**Conclusions:**

The EPS with a pharmacological autonomic blockade represents a useful approach for distinguishing extrinsic, functional bradycardia from intrinsic nodal disease in athletes. While AV node dysfunction appears exclusively vagally mediated and reversible, a subset of sinus node dysfunction cases may reflect early, possibly epigenetically driven, intrinsic alterations.

## 1. Introduction

Regular endurance training induces a range of structural and functional cardiovascular changes commonly referred to as “athlete’s heart”, including sinus bradycardia and atrioventricular (AV) conduction delay. These features are typically considered benign and attributed to increased vagal tone [1, 2]. According to international guidelines, even profound sinus bradycardia and first- or second-degree Mobitz I AV block may be considered physiological in asymptomatic athletes, provided that conduction normalizes during exertion [3]. However, several observational studies have raised concerns that these bradyarrhythmias may persist after detraining and could contribute to early sinus node (SN) or AV node dysfunction requiring pacemaker implantation, particularly in individuals with long-standing exposure to high-volume endurance training [4, 5].

While such changes are typically attributed to reversible vagal hyperactivity, emerging evidence suggests that in some athletes intrinsic adaptations of the cardiac conduction system may also play a role. These include long-term electrophysiologic remodeling and downregulation of ion channel gene expression, potentially mediated by epigenetic mechanisms [6, 7, 8]. However, clinical data confirming this hypothesis are scarce. To date, only two human studies have assessed intrinsic nodal function after pharmacologic autonomic blockade. The first, by Lewis *et al*. [9] in 1980, showed a significantly lower intrinsic heart rate in endurance-trained men compared to controls, suggesting a non-autonomic component to sinus bradycardia. More recently, Stein *et al*. [10] demonstrated that SN corrected recovery time (SNCRT) and AV nodal conduction remained prolonged in endurance athletes even after dual blockade, supporting the presence of intrinsic remodeling. That investigation involved only six endurance athletes, underscoring the need for larger studies to assess whether such intrinsic adaptations are prevalent or clinically relevant.

The present study aims to address this gap by evaluating intrinsic SN and AV node function in a cohort of elite athletes with bradycardia or conduction abnormalities using transesophageal electrophysiological study (EPS), both at baseline and after pharmacological autonomic blockade.

## 2. Methods

### 2.1 Study Population

The study sample of this retrospective observational study was derived from a cohort of 550 elite athletes enrolled at the Lesgaft National State University of Physical Education, Sports and Health (St. Petersburg, Russia) who underwent routine cardiovascular screening including history, physical examination, standard blood tests including thyroid function and electrolytes, 24-hour ambulatory ECG monitoring, maximal exercise testing and echocardiography. Among them, a subgroup, all engaged in mixed sports disciplines (involving both endurance and strength components) at national or international level, was referred for transesophageal EPS due to the presence of bradyarrhythmias or symptoms suggestive of arrhythmic disturbances. Those with suspected SN dysfunction (profound sinus bradycardia (<30 bpm), sinus pauses >3 seconds or second-degree sinoatrial block) or AV node dysfunction (advanced first degree AV block or daytime second degree Mobitz type I AV block) constituted the cases group, while those presenting with symptoms compatible with supraventricular tachyarrhythmias but without documented SN or AV node abnormalities formed the control group. All athletes had no history of structural heart disease, myocarditis, systemic conditions, or other known causes of secondary bradyarrhythmia. Data collection and electrophysiological studies were performed by the first author as part of a prior clinical and research activity at a former institution.

### 2.2 Electrophysiological Study Protocol

A non-invasive transesophageal EPS was performed in all participants to assess SN and AV node function. This technique, which provides reliable information on nodal conduction and refractoriness without the need for intracardiac catheterization, has been widely used in both clinical and research settings. A flexible multipolar electrode catheter was inserted transnasally into the esophagus and positioned at the level of the left atrium, guided by surface electrocardiographic (ECG) monitoring to ensure optimal atrial capture during pacing. Bipolar atrial stimulation was performed using rectangular pulses of 10 ms duration and twice diastolic threshold amplitude. Standard pacing protocols included incremental atrial pacing and extrastimulus testing to determine the corrected sinus node recovery time (CSNRT), the Wenckebach point, and the anterograde AV nodal effective refractory period (AVERP).

The CSNRT was measured as the interval between the last paced atrial stimulus and the first spontaneous SN depolarization, corrected by subtracting the basal sinus cycle length. A CSNRT >550 ms was considered abnormal. The Wenckebach point was defined as the shortest atrial pacing cycle length at which 1:1 AV conduction was no longer maintained, typically expected to be >350 ms in healthy individuals and considered prolonged when >500 ms. The AVERP was assessed by introducing progressively earlier premature atrial stimuli following a basic drive train of eight beats, and defined as the longest coupling interval failing to conduct to the ventricle.

Among the athletes in the case group, those who showed abnormal baseline values for either CSNRT or Wenckebach point underwent repeat EPS following pharmacological autonomic blockade. This blockade was achieved by sequential intravenous administration of atropine (0.04 mg/kg) to inhibit parasympathetic tone, followed by propranolol (0.2 mg/kg) to suppress sympathetic activity, according to the classic protocol described by Jose and Taylor. EPS parameters were re-assessed once heart rate stabilization confirmed effective autonomic suppression. No autonomic blockade was performed in the control group.

### 2.3 Statistical Analysis

Continuous variables are presented as mean ± standard deviation (SD) or as median with interquartile range, depending on their distribution, which was assessed using the Shapiro–Wilk test. Comparisons between the case and control groups, treated as independent samples, were performed using the Student’s *t*-test for normally distributed variables or the Mann–Whitney U test for non-normally distributed variables. Categorical variables were compared using the chi-square test or Fisher’s exact test, as appropriate. Within the subgroup of athletes who underwent EPS before and after autonomic blockade (n = 24), comparisons were made using paired tests for dependent samples. Specifically, the paired *t*-test was used for continuous variables with normal distribution, while the Wilcoxon signed-rank test was applied to non-parametric data. All statistical analyses were conducted using SPSS Statistics version 28.0 (IBM Corp., Armonk, NY, USA). A two-tailed *p*-value < 0.05 was considered statistically significant.

## 3. Results

### 3.1 Clinical Characteristics of the Study Population

The study population included 72 elite male athletes (mean age 20 ± 0.7 years) who had practiced mixed sports at national or international levels for an average of 10 ± 5 years. Among them, 58 athletes with suspected SN dysfunction or AV node constituted the case group, while 14 athletes with no evidence of bradyarrhythmias formed the control group. All participants were asymptomatic at rest or reported only mild symptoms during training, such as dizziness or fatigue. Blood test and exercise testing were normal (in particular, no chronotropic incompetence, AV conduction abnormalities or arrhythmias were recorded) and no structural cardiac abnormalities were detected on echocardiography. On 24-hour ECG monitoring, all abnormalities were observed at rest while no exercise-induced abnormalities were noted. Compared with controls, athletes in the case group demonstrated slightly larger left atrial area (2.0 ± 0.1 vs 1.8 ± 0.1 cm^2^/m^2^) and right ventricular diameter (1.7 ± 0.1 vs 1.3 ± 0.1 cm; *p* < 0.05). A significant correlation was found between the duration of training and the risk of atrial fibrillation induced during EPS (r = 0.3; *p* < 0.05) (Table 1).

**Table 1.** **Clinical and echocardiographic characteristics**.

	Case group (n = 58)	Control group (n = 14)	*p*-value
Age (years)	20 ± 0.7	20 ± 0.7	0.83
Years of training	10 ± 5	10 ± 5	0.72
Training volume (hours/week)	9.5 ± 5.2	9.3 ± 2.8	0.67
Resting HR (bpm)	34 ± 5.3	42 ± 6.1	<0.01
LA area (cm^2^/m^2^)	2.0 ± 0.1	1.8 ± 0.1	0.04
RV diameter (cm)	1.7 ± 0.1	1.3 ± 0.1	0.02
LV ejection fraction (%)	62.3 ± 8.3	58.7 ± 7.9	0.18
LV diastolic volume (mL)	62.3 ± 12.8	57.5 ± 11.2	0.24
LV systolic volume (mL)	22.8 ± 8.5	24.8 ± 7.9	0.50
Interventricular septal thickness (mm)	12.1 ± 4.1	11.6 ± 2.3	0.64
LV end-diastolic diameter (mm)	47.9 ± 6.1	49.6 ± 5.7	0.47
Posterior wall thickness (mm)	11.2 ± 2.2	10.7 ± 1.6	0.41
RV shortening fraction (%)	44.4 ± 4.8	44.4 ± 8.3	0.99
RV end-diastolic area (cm^2^)	23.3 ± 4.2	20.6 ± 5.4	0.31
RV end-systolic area (cm^2^)	14.0 ± 5.1	13.6 ± 6.0	0.87
TAPSE (mm)	24.0 ± 7.7	24.1 ± 5.2	0.97
Pulmonary artery pressure (mmHg)	21.3 ± 9.6	26.5 ± 9.3	0.22
IVC collapsibility index (%)	59.4 ± 7.3	59.4 ± 11.1	0.99

HR, heart rate; IVC, inferior vena cava; LA, left atrium; LV, left ventricle; RV, right ventricle; TAPSE, tricuspid annular plane systolic excursion.

### 3.2 Baseline Electrophysiological Study

At baseline, the CSNRT exceeded 550 ms in 18 (31%) of the case group, while the AV Wenckebach point was prolonged (>500 ms) in 8 (14%) of cases (553 ± 22 ms). In 12 athletes, supraventricular or ventricular ectopy was recorded, and atrial fibrillation was inducible in 11 (19%), all also with SN dysfunction, requiring pharmacologic cardioversion (Table 2).

**Table 2.** **Baseline electrophysiological study parameters**.

	Case group (n = 58)	Control group (n = 14)	*p*-value
CSNRT >550 ms, n (%)	18 (31%)	0	<0.001
Mean CSNRT (ms)	570 ± 20	420 ± 30	<0.001
Wenckebach point >500 ms, n (%)	8 (14%)	0	<0.01
Mean Wenckebach point (ms)	553 ± 22	420 ± 85	<0.01
Inducible AF, n (%)	11 (19%)	0	<0.001

AF, atrial fibrillation; CSNRT, corrected sinus node recovery time.

### 3.3 Electrophysiological Response to Autonomic Blockade

Of the 24 athletes with abnormal baseline EPS parameters (CSNRT >550 ms and/or Wenckebach cycle length >500 ms), all underwent repeat testing following complete pharmacologic autonomic blockade. After blockade, CSNRT remained prolonged (>550 ms) in 12/18 athletes with baseline prolonged CSNRT consistent with intrinsic SN dysfunction and normalized in the remaining 6 athletes. Notably, the heart rate before and after autonomic blockade remained comparable (34 ± 3.5 vs 34 ± 5.3 bpm).

**Fig. 1.**
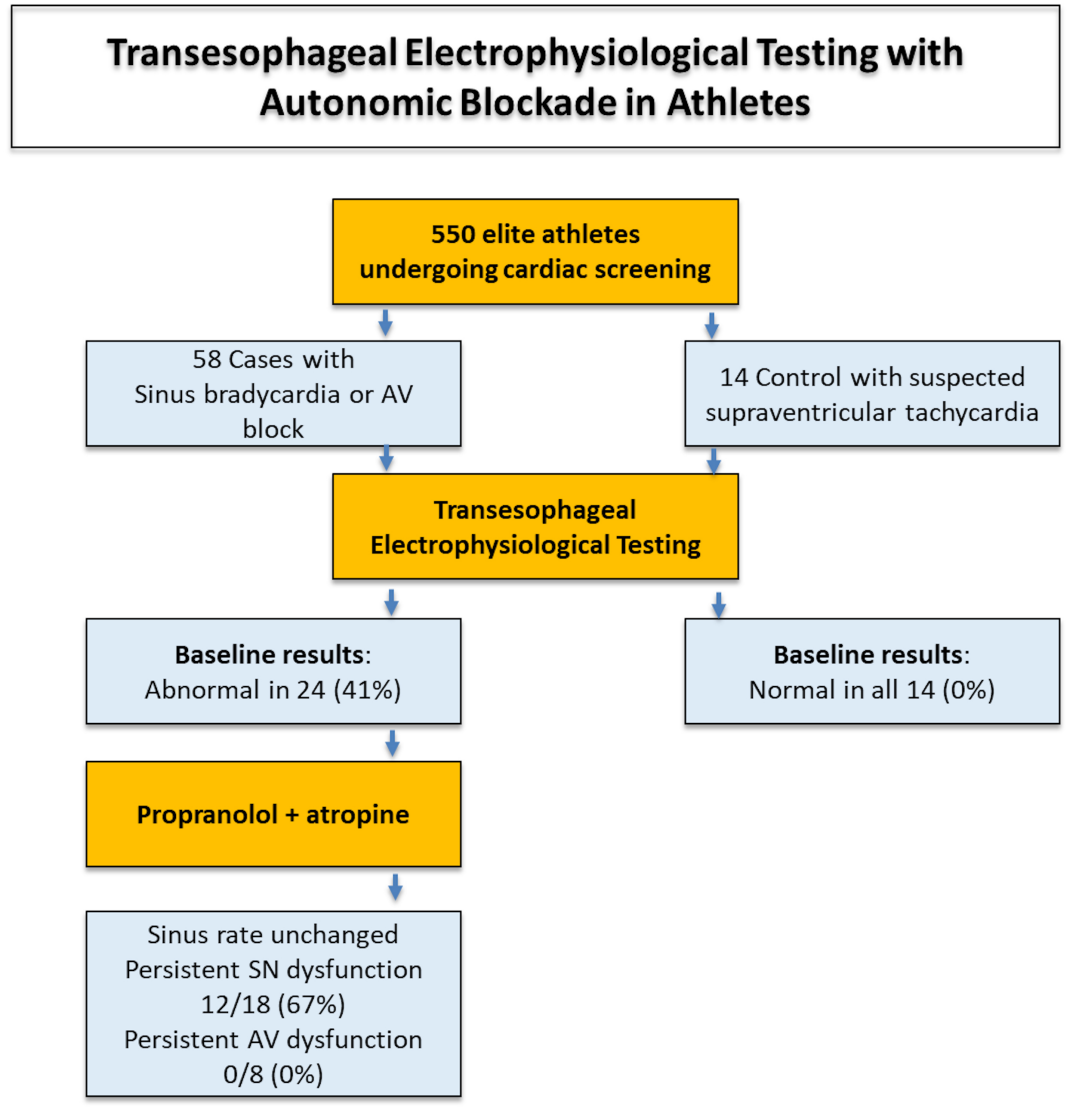
**Summary of the main study findings**. AV, atrioventricular; SN, sinus node.

AV nodal Wenckebach point normalized in all eight affected cases with baseline abnormalities consistent with extrinsic (neurally-mediated) dysfunction (Fig. 1). The AV refractory period also significantly improved (from 553 ± 22 ms to 443 ± 48 ms; *p* < 0.01), becoming comparable to control values (420 ± 85 ms) (Table 3).

**Table 3.** **Changes Before and After Autonomic Blockade in the subgroup of 24 athletes with abnormal baseline electrophysiological parameters**.

	Pre-blockade (n = 24)	Post-blockade (n = 24)	*p*-value
Heart rate (bpm)	34 ± 3.5	34 ± 5.3	0.71
CSNRT >550 ms, n (%)	18 (75%)	12 (50%)	0.07
Mean CSNRT (ms)	570 ± 20	500 ± 89	0.16
Wenckebach point >500 ms, n (%)	8 (33%)	0 (0%)	<0.01
Mean Wenckebach point (ms)	553 ± 22	443 ± 48	<0.01

CSNRT, corrected sinus node recovery time.

## 4. Discussion

**Fig. 2.**
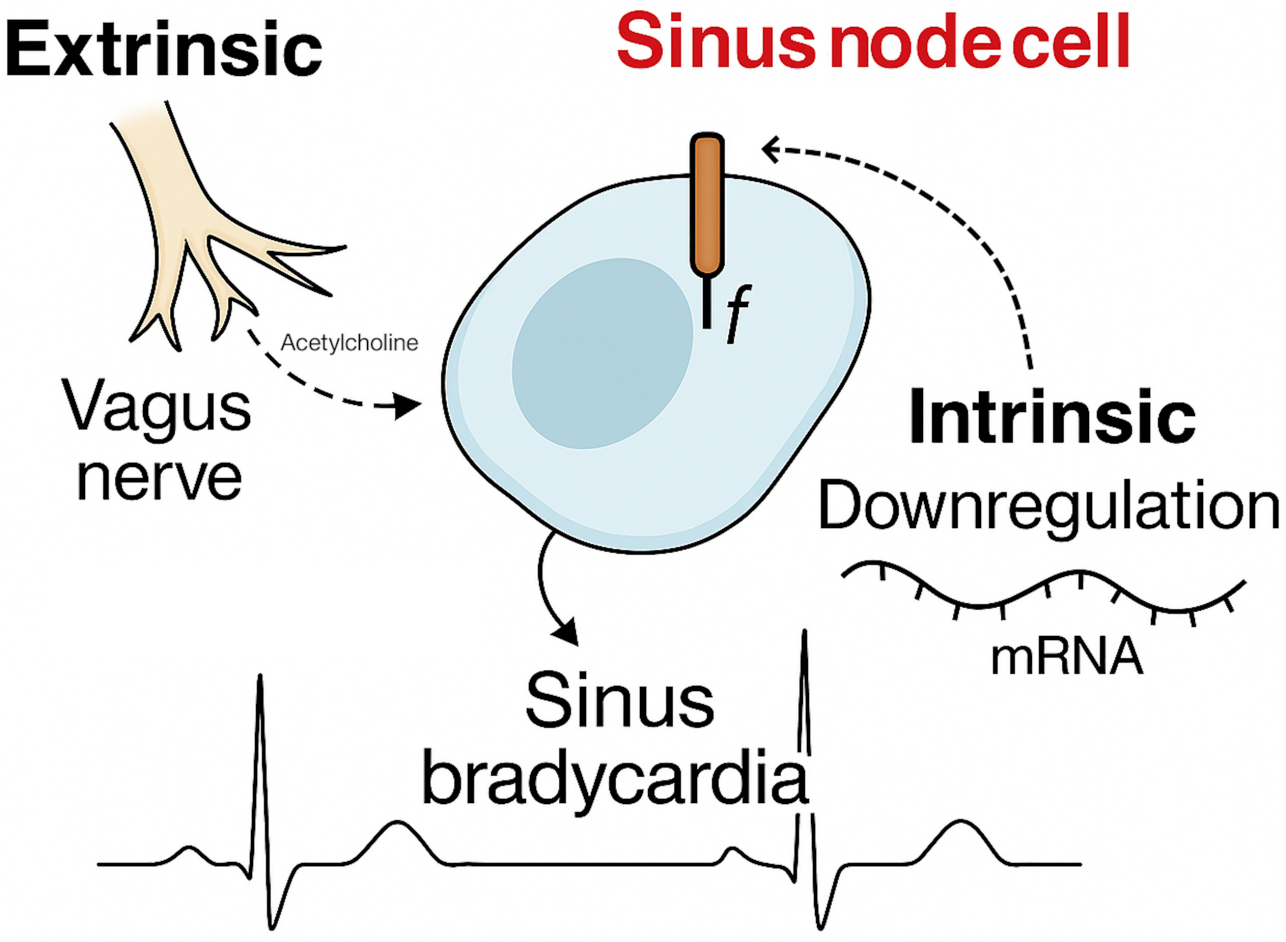
**Proposed mechanisms to explain sinus bradycardia in athletes**.

This study evaluated SN and AV node function in a cohort of elite athletes undergoing transesophageal EPS, both at baseline and after pharmacologic autonomic blockade. Among the 58 athletes with bradyarrhythmias or conduction disturbances, 24 underwent EPS with autonomic blockade due to abnormal baseline findings. While AV node abnormalities resolved completely after autonomic suppression, heart rate remained similar and two thirds of the athletes with baseline signs of SN dysfunction continued to exhibit prolonged CSNRT, suggesting an intrinsic mechanism. In contrast, all control athletes had normal EPS parameters without need for autonomic blockade. These results indicate that while AV node dysfunction in athletes is largely functional and reversible, a subset of SN disturbances may reflect structural or molecular remodelling (Fig. 2).

### 4.1 The Athlete’s Heart: Remodelling of the Cardiac Electrical System

Endurance training induces a wide range of cardiovascular adaptations collectively known as “athlete’s heart”. These include increased left ventricular volume, right ventricular enlargement, and biatrial dilation, typically accompanied by preserved or even enhanced systolic function [1, 2]. From an electrical perspective, sinus bradycardia and first-degree AV block are frequently observed and considered physiological [3]. Although these adaptations are attributed primarily to increased vagal tone and are considered benign and reversible with detraining, the distinction between adaptive changes and early signs of pathology can be subtle.

While most athletes demonstrate complete normalization of nodal function with exertion or pharmacological blockade, some may exhibit persistent conduction abnormalities that suggest the presence of maladaptive electrical remodeling. Observational data and emerging experimental evidence indicate that endurance training may, in select individuals, lead to sustained changes in ion channel expression and electrophysiological properties of the sinoatrial or AV nodes [4, 5, 6, 7]. This hypothesis is supported by the increased prevalence of symptomatic bradyarrhythmias and earlier need for pacemaker implantation observed in former athletes compared to sedentary individuals [4, 5, 11].

In a previous study from our group, former athletes implanted with pacemakers before the age of 70 for idiopathic SN or AV node dysfunction had a significantly lower mean age at implant than their non-athlete counterparts [11]. This difference was most pronounced in those with a history of high-volume endurance training, suggesting a dose-dependent relationship between training load and long-term conduction system remodelling. Similarly, Baldesberger *et al*. [4] reported higher rates of SN dysfunction in retired professional cyclists. Taken together, these findings raise the possibility that, in a subset of predisposed individuals, training-induced adaptations may become maladaptive and lead to clinically significant bradyarrhythmias in later life.

### 4.2 Physiology of Electrical Remodelling of the Athlete’s Heart: Extrinsic Versus Intrinsic Mechanisms

Traditionally, athlete’s bradycardia has been attributed almost exclusively to enhanced vagal tone, a benign and reversible extrinsic mechanism [1, 2]. This view is supported by observations that heart rate and AV conduction normalize during exercise and often return to baseline with detraining. Accordingly, international guidelines consider marked bradycardia or first-degree AV block in asymptomatic athletes as physiological if they resolve with exertion [3, 12]. However, several lines of evidence now challenge this paradigm, suggesting that intrinsic, possibly irreversible adaptations of the conduction system may also occur [13, 14, 15].

In animal models, prolonged exercise training leads to significant changes in SN function, including reduced expression of hyperpolarization-activated cyclic nucleotide-gated (HCN) channels, L-type calcium channels, and associated regulatory proteins [6, 7, 8]. These changes reduce automaticity and conduction velocity independent of autonomic tone. Translational studies have confirmed similar alterations in human athletes. For instance, Lewis *et al*. [9] demonstrated that SN recovery time and AV nodal conduction remained prolonged in six endurance athletes even after pharmacologic autonomic blockade. Additionally, Stein *et al*. [10] had already proposed in 2002 that intrinsic mechanisms may contribute to athlete’s bradycardia by showing a significantly lower intrinsic heart rate in endurance-trained individuals compared to controls following dual blockade. While their sample size was small, their findings suggested that training-induced electrophysiologic remodeling may not be entirely reversible.

Our study expands this previous observation by including a larger cohort and implementing a robust methodology based on noninvasive transesophageal EPS with dual autonomic blockade. Among the 24 athletes who underwent repeat EPS after blockade, all AV conduction abnormalities normalized, confirming their extrinsic nature. In contrast, CSNRT remained prolonged in 50% of athletes with initial SN dysfunction, indicating a persistent intrinsic abnormality. These findings confirm the presence of heterogeneous mechanisms underlying athlete’s bradycardia: while AV nodal delay is predominantly mediated by reversible vagal input, SN dysfunction may reflect a combination of vagal tone and intrinsic remodelling. However, no athlete demonstrated chronotropic incompetence during exercise testing or physical activity recorded by ambulatory ECG monitoring, suggesting that catecholamine stimulation is able to normalize the SN function.

### 4.3 Clinical Implications

The differentiation between extrinsic (vagal) and intrinsic (structural or molecular) nodal dysfunction is critical in sports cardiology: extrinsic bradycardia is benign and resolves with exertion or detraining while intrinsic dysfunction may progress to symptomatic disease requiring intervention [16]. Our data provide a practical electrophysiologic approach to this distinction. The normalization of EPS parameters after autonomic blockade supports a vagally mediated mechanism and favors conservative management. Conversely, persistent prolongation of CSNRT suggests a substrate that may not respond to detraining alone and may warrant closer follow-up.

These findings have implications for risk stratification and management in athletes with bradycardia or conduction disturbances. First, they support the use of EPS with via transesophageal approach (that is a non-invasive and widely available tool) with autonomic blockade in selected cases—particularly when symptoms such as syncope or presyncope occur, or when conduction abnormalities persist at rest. Second, they challenge the assumption that all bradyarrhythmias in athletes are functional. Third, they reinforce the importance of individualized evaluation: while most athletes can be safely reassured, a subset may harbor early conduction system disease.

From a long-term perspective, intrinsic SN dysfunction in athletes may contribute to the increased rate of pacemaker implantation observed in some cohorts of former endurance athletes [4, 5, 10]. Our previous studies suggest that a history of intense training may facilitate the age-related degenerative process affecting the conduction system [5, 10]. This highlights the need for follow-up in athletes with persistent bradycardia, especially if nodal recovery remains abnormal despite autonomic suppression.

### 4.4 Study Limitations

Several limitations must be acknowledged. First, the study cohort was relatively small, particularly the subgroup of athletes who underwent autonomic blockade, and consisted of Caucasian elite male athletes engaged in mixed sport disciplines. This limits the generalizability of our findings to other sport types/levels, gender and ethnic groups. Second, the number of athletes with baseline AV node dysfunction was low, limiting our ability to draw definitive conclusions about the intrinsic versus extrinsic nature of these conduction delays. Third, the lack of long-term follow-up prevents us from establishing the clinical course of intrinsic nodal dysfunction or its association with adverse outcomes. Finally, although echocardiographic data were available, we did not employ advanced imaging modalities such as cardiac magnetic resonance, which could provide further insights into subtle myocardial or conduction system abnormalities.

Despite these limitations, this is one of the largest human studies exploring intrinsic versus extrinsic mechanisms of bradycardia in athletes using direct electrophysiologic assessment and contributes important new data to a field previously supported primarily by animal models and anecdotal reports.

## 5. Conclusions

Sinus bradycardia and AV conduction delay are common findings in trained athletes and are usually considered benign, vagally mediated adaptations. However, our findings suggest that in a subset of athletes, particularly those with marked SN dysfunction, intrinsic remodelling may underlie persistent conduction abnormalities. These changes are not reversed by autonomic blockade and may indicate early nodal pathology. The proportion of athletes in whom this adverse nodal remodelling may become clinically relevant after cessation of an athletic career, potentially requiring pacemaker implantation, remains to be established.

## Availability of Data and Materials

Data supporting the article are available from the corresponding author upon reasonable request.
